# T2-weighted imaging hypointensity in an ovarian lesion: is it a benign finding?

**DOI:** 10.31744/einstein_journal/2022AO6851

**Published:** 2022-05-18

**Authors:** Juan Marcelo Fernandez Alcala, Thais Caldara Mussi, Luciana Cristina Pasquini Raiza, Ronaldo Hueb Baroni

**Affiliations:** 1 Hospital Israelita Albert Einstein São Paulo SP Brazil Hospital Israelita Albert Einstein, São Paulo, SP, Brazil.

**Keywords:** Ovarian neoplasms, Magnetic resonance imaging, Magnetic resonance spectroscopy, Algorithms

## Abstract

**Objective:**

To evaluate whether the presence of a hypointense signal at T2-weighted imaging in a solid ovarian lesion on magnetic resonance imaging is a predictor of stability and benignity.

**Methods:**

This is a single center study, prospectively read with retrospective acquired data. The database was searched for patients who underwent magnetic resonance imaging between January 2008 and October 2019 and whose reports mentioned solid ovarian lesions with low signal on T2-weighted imaging. A total of 47 nodules were included. A radiologist who was blinded to the clinical indication for magnetic resonance imaging and original reports evaluated the cases. Objective and subjective criteria of ovarian lesions in magnetic resonance imaging were evaluated.

**Results:**

Thirty-five nodules were considered benign/stable and 12 were considered non-stable. The analysis showed that the non-stable lesions showed statistically more hyperintensity at T1-weighted imaging compared to the stable lesions.

**Conclusion:**

T2-weighted imaging hypointensity can be considered a predictor of stability in solid ovarian lesions when associated with iso/hypointensity in T1-weighted imaging.

## INTRODUCTION

Neoplasms have a major impact on the morbidity and mortality and are currently the leading cause of death in most parts of the world.^( 1 )^ Some of the related risk factors of ovarian cancer are old age, family history, nulliparity or late parity, endometriosis, and BRCA mutation.^( 1 , 2 )^

Ovarian cancer is the seventh most common in women and the third most frequent among gynecologic neoplasms.^( 2 )^ Due to its usually silent and asymptomatic growth, the late onset of symptoms and the lack of an established screening program, it is usually diagnosed in advanced stages, with a high mortality rate.^( 2 )^

It is important to define whether an ovarian lesion is malignant or benign, with important clinical and therapeutic decision impact. If a newly detected ovarian lesion admits a substantial risk of malignancy, treatment should be performed at a specialist oncology center. Furthermore, most of the time they require surgery followed by chemotherapy or neoadjuvant chemotherapy. Women with benign adnexal masses may be treated conservatively or simply resecting the lesion. Thus, predictive models of malignant injury potential through imaging methods are constantly being developed to guide appropriate treatment and follow-up.^( 3 - 5 )^Differentiate benign and malignant lesions can avoid an unnecessary surgical procedure.^( 6 )^

In this context, magnetic resonance imaging (MRI) has proved to be a great method for identifying and characterizing ovarian lesions. Due to its high capacity for tissue differentiation, it is able to identify a component of hemorrhage, fat, and collagen (fibrosis). Fibrosis has low or intermediate signal intensity on T1-weighted (T1w) and low signal on T2-weighted (T2w) images.^( 7 )^ Many benign tumors, including teratoma, Brenner’s tumor, and stromal tumor, often show these characteristic features on MRI.^( 8 - 11 )^

Imaging findings predicting benignity in ovarian lesions, despite having great clinical value, often generate a dilemma in the differential diagnosis with solid malignant tumors, especially mixed/ solid-cystic lesions.^( 12 , 13 )^

## OBJECTIVE

To evaluate whether the presence of hypointense signal intensity on the T2-weighted imaging magnetic resonance imaging sequence in an ovarian lesion can be related with benignity or stability.

## METHODS

This is a single-center study, prospectively read with retrospective data acquired at a private tertiary hospital, approved with waiver for informed consent in the Institutional Review Board.

A database was searched to select patients who performed MRI between January 2008 and October 2019, and whose MRI reports mentioned solid low signal lesions on T2w images.

The search for the cases was done through a keyword search system in the reports (BI system). The terms we used were: “ovarian lesion”, “ovarian nodule”, “hypointense on T2w sequences”, “low-intensity signal on T2”, “Brenner”, “fibroma”, “fibrothecoma”, “fibrous” and “stromal”.

**Figure 1:**
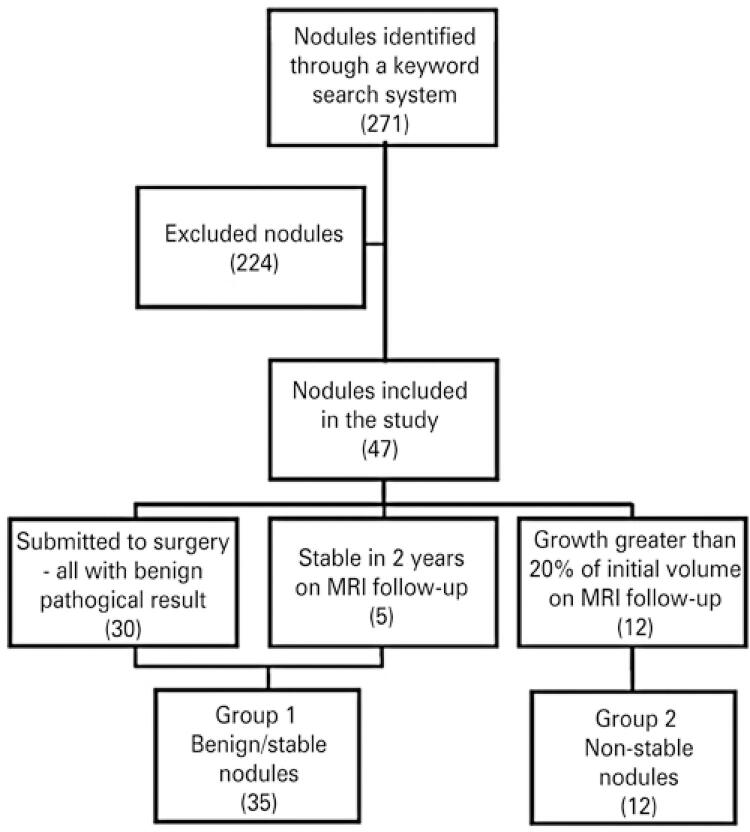
Flowchart shows the formation of Groups 1 and 2 included in the study

A total of 271 cases were identified. Of these, 224 cases were excluded due to error in medical record number (n=1), absence of anatomopathological results at our institution and less than 2 years in follow-up evaluations (n=96), error/confusion in the identification of keywords (n=115), absence of images available on our system (n=1), repetition of the case in search (n=8), and non-lesions characterization on follow-up test (n=3). With this, a total of 47 lesions in 41 patients were included in this study ( Figure 1 ). All of these 47 ovarian lesions were entirely solid, with no cystic component associated.

MRI: magnetic resonance imaging.

The exams were performed in both 1.5 and 3 Tesla scanner in 1 of the 8 scanners available: Magnetom Prisma, Aera, Espree (Siemens Medical Solutions USA, Malvern, PA, USA), Discovery MR 750W, Signa 450W, Signa Artist, HDxt (GE Healthcare, Little Chalfont, United Kingdom) and Ingenia (Philips Research, Eindhoven, Netherlands). A routine protocol included coronal 3D isotropic T2w imaging, axial and sagittal fast spin echo (FSE) T2w imaging, axial T1w imaging in and out-of-phases, axial diffusion-weighted imaging (DWI), apparent diffusion coefficient (ADC) map, axial and sagittal pre contrast T1w imaging, arterial, venous and delayed sagittal T1w imaging post contrast, and axial delayed post contrast phases.

One of the authors anonymized the selected cases that were evaluated on a Picture Archiving and Communication System PACS (PACS ) workstation (KODAK/Carestream; Carestream Health, Rochester, New York, USA) by a radiologist with 10 years of experience in abdominal radiology who was blinded to clinical MRI indication and original reports. Objective and subjective MRI criteria of solid ovarian lesions were evaluated. Objective criteria included number of lesions, dimensions and laterality. Subjective criteria included contours, localization in the ovary (central or cortical), homogeneity on T2w imaging, signal intensity on T1w imaging (compared to an ipsilateral pelvic muscle), presence of diffusion restriction and pattern of contrast enhancement (compared with the contralateral ovarian parenchyma). The assessment of signal intensity on T1w imaging was subjective and objective, with measurement of a region of interest (ROI) in at least 50% of the ovarian lesion compared to a pelvic ipsilateral muscle in the same image of the lesion (avoiding muscles with fat substitution), in general the internal obturator muscle or iliac muscle.

The gold standard used to characterize a lesion as benign was anatomopathological result or lesion stability on MRI follow-up for at least 2 years (Group 1). Lesions with histopathological results compatible with malignancy (in cases submitted to surgery) and lesions that grew in dimensions (at least 20% of the initial volume) on MRI follow-up in at least 2 years were considered non-stable (Group 2). Quantitative and qualitative characteristics were described in ovarian lesions of Groups 1 and 2. These characteristics were also compared between these groups, comparing stable lesions (Group 1) with non-stable lesions (Group 2).

Quantitative variable was described in mean, standard deviation, median and interquartile interval and were compared with *t-* Student test. Qualitative variables (contours, homogeneity, presence of diffusion restriction, and presence and intensity of contrast enhancement) were described according to the criterion of benign/non-stable using absolute and relative frequencies, and the association verified with the use of exact tests (Fisher’s exact test or likelihood ratio test).

A joint model was constructed to evaluate the influence of characteristics on benign/non-definitively benign using a multiple logistic regression model. The analyses were performed using the software SPSS for Windows, version 22.0. The level for statistical significance was set at 5%.

The study was approved by the Research Ethics Committee of the *Hospital Israelita Albert Einstein* (HIAE) (CAAE: 26632819.9.0000.0071, report # 3.952.640). Institutional Review Board approved this study with waiver for informed consent.

## RESULTS

Of the 47 nodules, 30 underwent surgery and had pathological results. The 17 remaining nodules had MRI control for at least 2 years and no histological results confirmation.

Of the 30 nodules that underwent surgery, all had benign pathological results, of which 19 (63.3%) were fibroids, 5 (16.7%) were Brenner tumors, 2 (6.7%) were leiomyomas (1 of uterine tube), 1 (3.3%) was fibrothecoma, 1 (3.3%) was ligamentous fibrous nodule, 1 (3.3%) was serous cystadenofibroma, and 1 (3.3%) was endometrioid adenofibroma.

Of the 17 nodules that had MRI control of at least 2 years (not submitted to surgery), only 5 remained stable, presented no growth or growth below 20% comparing to the initial volume. The remaining 12 nodules grew more than 20% and were considered non-stable, as shown in table 1 .

**Table 1:** Comparison of the evaluated features between groups

Variable	Group 1	Group 2	Total	p value
Size of the lesion (mm- long axis)				0.220^#^
mean±SD	28.1±15.2	22.3±9.1	26.6±14	
median (min-max)	25 (10-82)	20 (14-46)	24 (10-82)	
Size of the lesion (mm)				0.140^#^
mean±SD	22.7±12.4	17.1±5.3	21.2±11.2	
median (min-max)	20 (9-65)	16.5 (11-29)	18 (9-65)	
Size of the lesion (mm)				0.173^#^
mean±SD	19.3±11.6	14.4±5.8	18±10.6	
median (min-max)	16 (8-58)	14 (8-26)	16 (8-58)	
Localization, n (%)				0.713*
Cortical/peripheral	26 (74.3)	8 (66.7)	34 (72.3)	
Medullary/central	9 (25.7)	4 (33.3)	13 (27.7)	
Signal in T1w, n (%)				0.006^†^
Isosignal	12 (34.3)	1 (8.3)	13 (27.7)	
Hyposignal	12 (34.3)	1 (8.3)	13 (27.7)	
Hypersignal	11 (31.4)	10 (83.3)	21 (44.7)	
Homogeneity in T2w, n (%)				0.471*
Homogeneous	27 (77.1)	8 (66.7)	35 (74.5)	
Heterogeneous	8 (22.9)	4 (33.3)	12 (25.5)	
Contours, n (%)				0.205*
Regular	30 (85.7)	8 (66.7)	38 (80.9)	
Irregular	5 (14.3)	4 (33.3)	9 (19.1)	
DWI, n (%)				0.386^†^
Absent	20 (60.6)	6 (54.5)	26 (59.1)	
Mild restriction	7 (21.2)	1 (9.1)	8 (18.2)	
Moderate/severe restriction	6 (18.2)	4 (36.4)	10 (22.7)	
Pattern of enhancement, n (%)				0.109^†^
Hypovascular	16 (51.6)	7 (87.5)	23 (59)	
Isovascular	4 (12.9)	0 (0)	4 (10.3)	
Hypervascular	11 (35.5)	1 (12.5)	12 (30.8)	

* Fisher’s exact test; ^#^ *t* -Student test; ^†^ likelihood ratio test.

SD: standard deviation; T1w: T1-weighted; T2w: T2-weighted; DWI: diffusion-weighted imaging; min-max: minimum-maximum.

Thus, 35 nodules were considered benign or stable (Group 1), 30 due to pathological results and 5 due to follow-up imaging exams. Twelve nodules were included in non-stable lesions due to the growth greater than 20% of their initial volume in follow-up imaging exams (Group 2).

Lesions were measured in 3 orthogonal axes, with a mean size of 21.9mm (range 8-82mm) and median size of 19mm.

Regarding ovary localization, 26 (74.3%) lesions of Group 1 and 8 (66.7%) of Group 2 were cortical/peripheral and 9 (25.7%) of Group 1 and 4 (33.3%) of Group 2 were central (p=0.713). Contours were regular in 30 (85.7%) lesions of Group 1 and 8 (66.7%) nodules of Group 2 and irregular in 5 (14.3%) lesions in Group 1 and 4 (33.3%) in Group 2 (p=0.205).

On T2w images, 27 lesions (77.1%) of Group 1 had homogeneous signal intensity and 8 (22.9%) had heterogeneous signal intensity; and Group 2 had 8 (66.7%) lesions with homogeneous signal intensity and 4 nodules (33.3%) with heterogeneous signal intensity (p=0.471).

**Figure 2:**
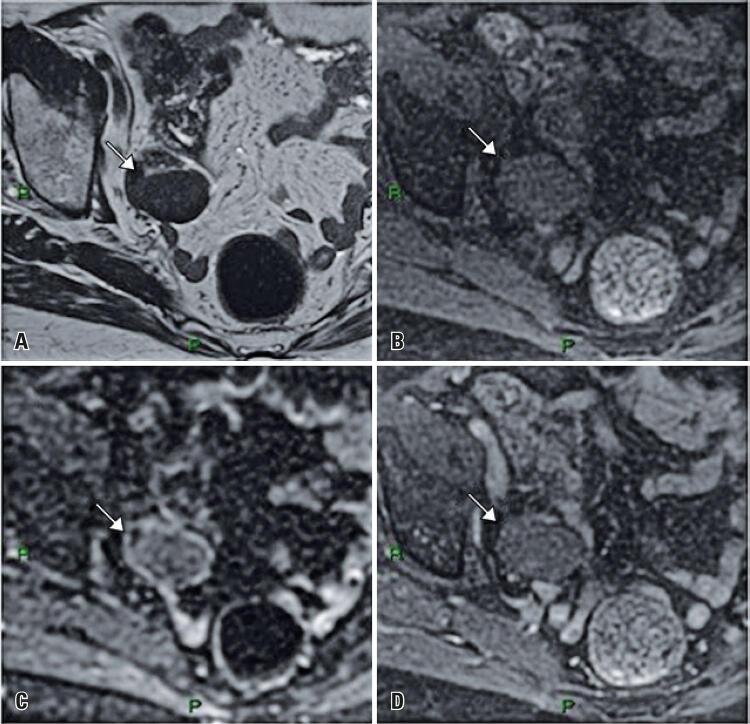
Images from an 84-year-old woman. Images show hyposygnal nodule in right ovary on T2-weighted imaging (A) and on fat-saturated T1-weighted imaging (B), with no water restriction on diffusion-weighted imaging (C) and with hypovascular enhancement after intravenous contrast injection (D). The lesion was surgically resected and confirmed as fibroma (included in Group 1 of this study)

**Figure 3:**
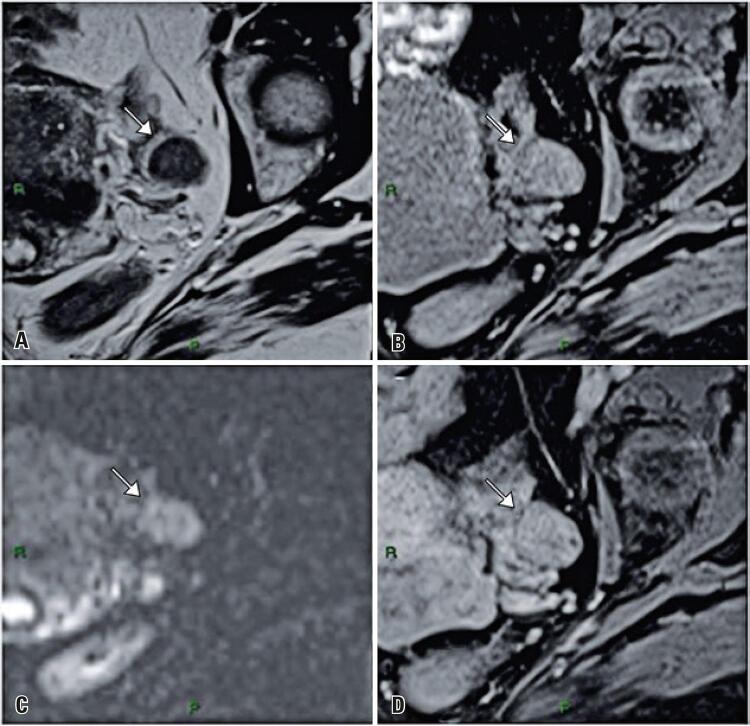
Images from a 53-year-old woman. Images show hyposygnal nodule in right ovary on T2-weighted imaging (A) and hypersignal on fat-saturated T1-weighted imaging (B), with mild water restriction on diffusion-weighted imaging (C) and with isovascular enhancement after intravenous contrast injection (D). The lesion showed growth greater than 20% on follow-up magnetic resonance imaging after 6 years and non-surgically resected (included in Group 2 of this study)

The only variable that had statistical differences comparing both groups were signal on T1w images, comparing with the muscle (p=0.006): in Group 1, 11 (31.4%) lesions had hyperintensity, 12 (34.3%) had hypointensity and 12 (34.3%) had isointensity. And in Group 2, 10 (83.3%) had hyperintensity, 1 (8.3%) had hypointensity and 1 (8.3%) had isointensity ( Figures 2 and 3 ).

On DWI and ADC map, 3 exams had this sequence not available. In Group 1, 20 (60.6%) lesions had no water diffusion restriction, 7 (21.2%) had mild diffusion restriction and 6 (18.2%) had moderate/severe diffusion restriction. In Group 2, 6 (54.5%) lesions had no water diffusion restriction, 1 (9.1%) had mild diffusion restriction and 4 (36.4%) had moderate/severe diffusion restriction (6 of Group 1 and 4 of Group 2), (p=0.386).

When compared with the contralateral ovarian parenchyma, the pattern of lesions enhancement was mostly hypovascular in both groups. Eight tests had no post-contrast sequences. In Group 1, 16 (51.6%) lesions were hypovascular, 4 (12.9%) were isovascular and 11 (35.5%) were hypervascular. In Group 2, 7 (87.5%) lesions were hypovascular and 1 (12.5%) was hypervascular (p=0.109).

## DISCUSSION

The analysis of the characteristics between both groups showed that the only lesion feature statistically significant different between Groups 1 and 2 was hyperintensity at T1w images (more frequent in Group 2 non-stable lesions). Therefore, solid ovarian lesions with hypointensity on T2w images and hyperintensity on T1w images in our study tended to grow in control exams (not stable). All other features evaluated had no statistically significant difference between groups.

Among the analyzed characteristics, T1w images signal was the only one that presented a significant statistical difference, with the non-stable lesions group presenting as hyperintense on this MRI sequence. Benign (stable) lesions, particularly fibrous (more common), tend to have a low T1w signal intensity.^( 8 , 9 , 12 , 13 )^ Hyperintensity in T1w is a broad group and may be present in benign and malignant ovarian lesions. The hyperintensity in T1w images may be due to fat, blood products or protein/mucinous component. Fat suppressed T1w and post-contrast subtraction images are required to characterize the component of these lesions and guide the diagnosis.^( 14 )^

Of the 47 nodules with low signal on T2w images included in this study, the majority, 35 (74.5%) were proven to be benign/stable, which is consistent with several studies in the literature.^( 8 , 7 , 15 , 16 )^ The remaining 12 nodules were characterized as non-stable, as they presented growth over 20% in the control exams within 2 years, however these nodules cannot be classified as malignant. Three nodules of those submitted to surgery also showed growth in previous control exams, being operated later, with a benign anatomopathological result. Therefore, the growth of a nodule cannot be definitive of a malignant lesion, since even benign lesions such as fibromas can grow over the years.

Probably the numerical (n) difference between Groups 1 and 2 in this study is due the fact that these lesions are mostly stable and being followed up rather than undergoing surgical resection.

The present study showed no significant difference regarding the presence and degree of diffusion or the enhancement pattern. The study of Thomassin-Naggara et al.^( 17 )^ showed that diffusion and perfusion images increase the diagnostic accuracy of a complex adnexal lesion, distinguishing benign from malignant lesions. It is important to mention that this study separated benign/stable lesions from non-stable lesions, not benign from malignant lesions.

The Ovarian-Adnexal Reporting and Data System (O-RADS) proposed by the American College of Radiology (ACR) uses MRI imaging features for the classification of malignant risk of ovarian lesions. The appearance of ovarian solid lesions on T2w images is 1 of the features that this system uses for the risk classification. Solid ovarian lesions with homogenous hypointensity are classified as almost certainly benign, with a risk of malignancy less than 0.5%, thus congruent with the findings of our study.^( 18 )^

Most lesions evaluated in this study (59%) had hypovascular pattern of enhancement, in both groups. Siegelman et al.^( 7 )^ showed that fibromas and fibrothecomas were characterized with low intensity on T2w and T1w signal. These lesions were also characterized as being hypovascular due to their fibrous character.

This study has some limitations. Only one radiologist read the cases; hence, interobserver agreement was not evaluated. The variability of magnetic resonance devices (a total of eight), both 1.5T and 3T, which performed the images included in the study, with consequent variability of signal and contrast on the images. The numerical difference between the Groups 1 and 2. In addition to, most importantly, absence of anatomopathological results in some lesions included, so we do not have a group comparison of benign and malignant lesions (we had no confirmation of malignant lesions), however we considered them in a different group of non-definitively benign lesions (non-stable lesions). We emphasize the need of multicentric studies with a more casuistic/ovarian lesions for the validation of the results.

## CONCLUSION

The results of this study found that hypointensity on T2-weighted imaging in an ovarian solid lesion can be considered as predictor of benignity and stability when associated with iso and hypointensity on T1-weighted imaging.
